# Poly[bis­(cyanato-κ*N*)bis­(μ-pyrazine-κ^2^ *N*:*N*′)cobalt(II)]

**DOI:** 10.1107/S1600536809005686

**Published:** 2009-03-25

**Authors:** Mario Wriedt, Inke Jess, Christian Näther

**Affiliations:** a Institut für Anorganische Chemie, Christian-Albrechts-Universität Kiel, Max-Eyth-Strasse 2, D-24118 Kiel, Germany

## Abstract

In the crystal structure of the title compound, [Co(NCO)_2_(C_4_H_4_N_2_)_2_]_*n*_, the Co(II) cation is coordinated by four *N*-bonded pyrazine ligands and two *N*-bonded cyanate anions in a slightly distorted octa­hedral geometry. The crystal structure consists of μ-*N*:*N*′ pyrazine-bridged cobalt cyanate chains; these are further linked by additional μ-*N*:*N*′-bridging pyrazine ligands into layers, which are stacked perpendicular to the crystallographic *a* axis. The C and O atoms in both crystallographic independent cyanate anions are disordered in two orientations and were refined using a split model with site occupation factor ratios of 0.75/0.25 and 0.7/0.3.

## Related literature

For related pyrazine structures, see: Lloret *et al.* (1999 ▶); Real *et al.* (1991 ▶); Lu *et al.* (1997 ▶); Wriedt *et al.* (2009 ▶). For general background, see: Näther & Greve (2003 ▶); Näther *et al.* (2003 ▶); Wriedt *et al.* (2008 ▶, 2009 ▶); Näther *et al.* (2007 ▶); Näther & Jess (2004 ▶).
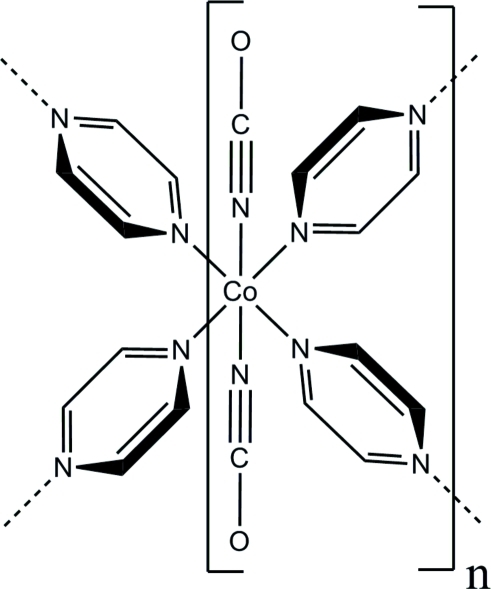


## Experimental

### 

#### Crystal data

[Co(NCO)_2_(C_4_H_4_N_2_)_2_]
*M* *_r_* = 303.15
Monoclinic, 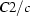

*a* = 25.5712 (17) Å
*b* = 10.1230 (8) Å
*c* = 10.1863 (7) Å
β = 104.763 (8)°
*V* = 2549.8 (3) Å^3^
*Z* = 8
Mo *K*α radiation
μ = 1.35 mm^−1^
*T* = 170 K
0.24 × 0.14 × 0.07 mm

#### Data collection

Stoe IPDS-1 diffractometer
Absorption correction: numerical (*X-SHAPE* and *X-RED32*; Stoe & Cie, 2008 ▶) *T*_min_ = 0.789, *T*_max_ = 0.903
11369 measured reflections
2684 independent reflections
2058 reflections with *I* > 2σ(*I*)
*R*_int_ = 0.039

#### Refinement

*R*[*F* ^2^ > 2σ(*F* ^2^)] = 0.051
*wR*(*F* ^2^) = 0.139
*S* = 1.04
2684 reflections
198 parameters
H-atom parameters constrained
Δρ_max_ = 0.71 e Å^−3^
Δρ_min_ = −1.17 e Å^−3^

### 

Data collection: *X-AREA* (Stoe & Cie, 2008 ▶); cell refinement: *X-AREA*; data reduction: *X-AREA*; program(s) used to solve structure: *SHELXS97* (Sheldrick, 2008 ▶); program(s) used to refine structure: *SHELXL97* (Sheldrick, 2008 ▶); molecular graphics: *XP* in *SHELXTL* (Sheldrick, 2008 ▶); software used to prepare material for publication: *XCIF* in *SHELXTL*.

## Supplementary Material

Crystal structure: contains datablocks I, global. DOI: 10.1107/S1600536809005686/bt2872sup1.cif

Structure factors: contains datablocks I. DOI: 10.1107/S1600536809005686/bt2872Isup2.hkl

Additional supplementary materials:  crystallographic information; 3D view; checkCIF report

## Figures and Tables

**Table 1:** Selected geometric parameters (Å, °)

Co1—N21	2.039 (3)
Co1—N31	2.059 (3)
Co1—N11	2.191 (3)
Co1—N2^i^	2.193 (3)
Co1—N12^ii^	2.197 (3)
Co1—N1	2.200 (3)

Symmetry codes: (i) 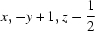; (ii) 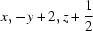.

**Fig. 1.**
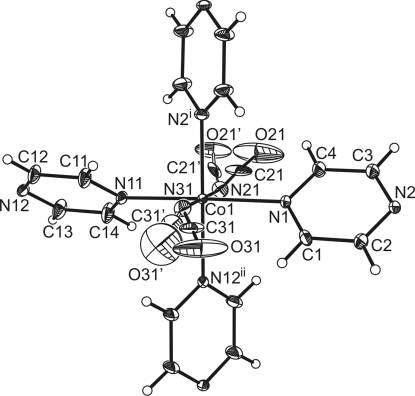
Crystal structure of the title compound with labelling and displacement ellipsoids drawn at the 50% probability level. The disorder of the cyanate anions is not show for clarity. [Symmetry codes: (i) x, -y+1, z-1/2; (ii) x, -y+2, z+1/2.]

**Fig. 2.**
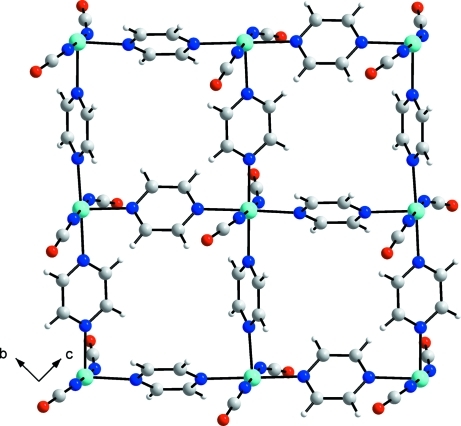
Crystal structure of the title compound with view along the a-axis.

## supplementary crystallographic information

### Comment

In our investigations we have recently demonstrated that new ligand deficient coordination polymers with interestic magnetic properties can be prepared by thermal decomposition of suitable ligand rich precursor compounds (Näther & Greve, 2003; Wriedt *et al.*, 2009). In order to prepare additional ligand rich precursor compounds we have reacted cobalt(II) nitrate hexahydrate and potassium cyanate with pyrazine in a methanol water mixture. In this reaction single crystals of the title compound were obtained in an inhomogenous mixture containing additional unknown phases.

In the crystal structure of the 1:2 title compound [Co(OCN)_2_(pyrazine)_2_]_n_ the cobalt(II) cations are coordinated by four pyrazine ligands and two thiocyanate anions within slightly distorted octahedra (Fig. 1). The cobalt cations are µ-1,4-(*N*,*N*) bridged by the pyrazine ligands forming layers, which are stacked perpendicular to the crystallographic *a-*axis (Fig. 2). The cyanate anions do not act as bridging ligands and are only terminal *N*-bonded to the metal center. A similar structural motif is observed in the structures of the 1:2 thiocyanate compounds of composition [M(SCN)_2_(pyrazine)_2_]_n_ (M = Mn, Fe, Co, Ni) reported recently (Lloret *et al.*, 1999; Real *et al.*, 1991; Lu *et al.*., 1997; Wriedt *et al.*, 2009). The Co—NCO distances amount to 2.039 (3) and 2.059 (3) Å and are significantly shorter as the Co—N_pyrazine_ distances, which range from 2.191 (3) to 2.200 (3) Å. The angles around the cobalt cations range between 89.23 (12) and 179.58 (12)° (Tab. 1). The shortest intra- and interchain Co···Co distances amount to 7.1721 (4) and 8.2170 (5) Å, respectively.

### Experimental

Co(NO_3_)_2_.6H_2_O, pyrazine and methanol were obtained from Alfa Aesar as well as KOCN was obtained from Fluka. 0.5 mmol (145.5 mg) Co(NO_3_)_2_.6H_2_O, 1 mmol (81.1 mg) KOCN, 0.5 mmol (40.1 mg) pyrazine, 0.5 mL methanol and 0.5 mL water were transfered in a closed test-tube. The mixture was heated at 120 °C for three days. After cooling yellow block-shaped single crystals of the title compound were obtained in a heterogenous mixture.

### Refinement

All H atoms were located in a difference map but they were positioned with idealized geometry and were refined with *U*_eq_(H) = 1.2 *U*_eq_(C) using a riding model with C—H = 0.95 Å.

The C and O atoms in both crystallographic independent cyanate anions are disordered in two orientations and were refined using a split model. In one of the two anions, the C and O atoms of lower occupancy were refined only isotropically. They were refined by a split model with site occupation factor ratios of 0.75/0.25 and 0.7/0.3.

### Crystal data

**Table:**

[Co(NCO)_2_(C_4_H_4_N_2_)_2_]	*F*(000) = 1224
*M**_r_* = 303.15	*D*_x_ = 1.579 Mg m^−^^3^
Monoclinic, *C*2/*c*	Mo *K*α radiation, λ = 0.71073 Å
Hall symbol: -C 2yc	Cell parameters from 8000 reflections
*a* = 25.5712 (17) Å	θ = 14.1–25.9°
*b* = 10.1230 (8) Å	µ = 1.35 mm^−^^1^
*c* = 10.1863 (7) Å	*T* = 170 K
β = 104.763 (8)°	Block, yellow
*V* = 2549.8 (3) Å^3^	0.24 × 0.14 × 0.07 mm
*Z* = 8	

### Data collection

**Table:**

Stoe IPDS-1 diffractometer	2684 independent reflections
Radiation source: fine-focus sealed tube	2058 reflections with *I* > 2σ(*I*)
graphite	*R*_int_ = 0.039
φ scans	θ_max_ = 27.1°, θ_min_ = 2.9°
Absorption correction: numerical (*X-SHAPE* and *X-RED32*; Stoe & Cie, 2008)	*h* = −32→32
*T*_min_ = 0.789, *T*_max_ = 0.903	*k* = −12→12
11369 measured reflections	*l* = −13→13

### Refinement

**Table:**

Refinement on *F*^2^	Primary atom site location: structure-invariant direct methods
Least-squares matrix: full	Secondary atom site location: difference Fourier map
*R*[*F*^2^ > 2σ(*F*^2^)] = 0.051	Hydrogen site location: inferred from neighbouring sites
*wR*(*F*^2^) = 0.139	H-atom parameters constrained
*S* = 1.04	*w* = 1/[σ^2^(*F*_o_^2^) + (0.0575*P*)^2^ + 18.631*P*] where *P* = (*F*_o_^2^ + 2*F*_c_^2^)/3
2684 reflections	(Δ/σ)_max_ = 0.001
198 parameters	Δρ_max_ = 0.71 e Å^−^^3^
0 restraints	Δρ_min_ = −1.17 e Å^−^^3^

### Special details

**Table:**

Geometry. All e.s.d.'s (except the e.s.d. in the dihedral angle between two l.s. planes) are estimated using the full covariance matrix. The cell e.s.d.'s are taken into account individually in the estimation of e.s.d.'s in distances, angles and torsion angles; correlations between e.s.d.'s in cell parameters are only used when they are defined by crystal symmetry. An approximate (isotropic) treatment of cell e.s.d.'s is used for estimating e.s.d.'s involving l.s. planes.
Refinement. Refinement of *F*^2^ against ALL reflections. The weighted *R*-factor *wR* and goodness of fit *S* are based on *F*^2^, conventional *R*-factors *R* are based on *F*, with *F* set to zero for negative *F*^2^. The threshold expression of *F*^2^ > σ(*F*^2^) is used only for calculating *R*-factors(gt) *etc*. and is not relevant to the choice of reflections for refinement. *R*-factors based on *F*^2^ are statistically about twice as large as those based on *F*, and *R*- factors based on ALL data will be even larger.

### Fractional atomic coordinates and isotropic or equivalent isotropic displacement parameters (Å^2^)

**Table:**

	*x*	*y*	*z*	*U*_iso_*/*U*_eq_	Occ. (<1)
Co1	0.629290 (19)	0.75059 (4)	0.63502 (4)	0.01326 (17)	
N1	0.62838 (13)	0.5943 (3)	0.7846 (3)	0.0162 (6)	
C1	0.66193 (17)	0.5983 (3)	0.9072 (4)	0.0224 (8)	
H1	0.6868	0.6695	0.9297	0.027*	
C2	0.66194 (17)	0.5014 (4)	1.0044 (3)	0.0224 (8)	
H2	0.6864	0.5088	1.0916	0.027*	
N2	0.62829 (13)	0.3981 (3)	0.9778 (3)	0.0171 (6)	
C3	0.59444 (17)	0.3935 (4)	0.8537 (3)	0.0230 (8)	
H3	0.5700	0.3215	0.8305	0.028*	
C4	0.59412 (17)	0.4920 (4)	0.7578 (4)	0.0225 (8)	
H4	0.5690	0.4864	0.6714	0.027*	
N11	0.63140 (13)	0.9093 (3)	0.4902 (3)	0.0163 (6)	
C11	0.67047 (17)	0.9151 (4)	0.4247 (4)	0.0228 (8)	
H11	0.6992	0.8528	0.4461	0.027*	
C12	0.67008 (17)	1.0102 (3)	0.3260 (4)	0.0222 (8)	
H12	0.6984	1.0110	0.2810	0.027*	
N12	0.63094 (13)	1.1005 (3)	0.2930 (3)	0.0167 (6)	
C13	0.59214 (17)	1.0958 (4)	0.3588 (4)	0.0259 (9)	
H13	0.5637	1.1590	0.3381	0.031*	
C14	0.59239 (18)	1.0000 (4)	0.4575 (4)	0.0259 (9)	
H14	0.5641	0.9993	0.5025	0.031*	
N21	0.54684 (14)	0.7557 (3)	0.5817 (3)	0.0238 (7)	
C21	0.5047 (3)	0.7083 (8)	0.5445 (8)	0.0347 (16)	0.75
O21	0.4602 (3)	0.6596 (9)	0.5110 (10)	0.109 (4)	0.75
O21'	0.4627 (6)	0.7506 (17)	0.4184 (18)	0.052 (4)	0.25
C21'	0.5054 (9)	0.7583 (16)	0.498 (2)	0.023 (4)	0.25
N31	0.71251 (14)	0.7443 (3)	0.6873 (3)	0.0218 (7)	
C31	0.7539 (3)	0.7288 (7)	0.7698 (8)	0.0250 (15)	0.70
O31	0.7962 (3)	0.7135 (8)	0.8513 (7)	0.074 (3)	0.70
C31'	0.7530 (9)	0.7621 (19)	0.734 (2)	0.030 (6)*	0.30
O31'	0.8018 (11)	0.771 (2)	0.789 (3)	0.098 (8)*	0.30

### Atomic displacement parameters (Å^2^)

**Table:**

	*U*^11^	*U*^22^	*U*^33^	*U*^12^	*U*^13^	*U*^23^
Co1	0.0272 (3)	0.0060 (2)	0.0076 (2)	−0.00033 (18)	0.00627 (18)	0.00004 (16)
N1	0.0307 (18)	0.0095 (13)	0.0085 (13)	−0.0035 (11)	0.0050 (13)	0.0015 (10)
C1	0.037 (2)	0.0124 (16)	0.0150 (16)	−0.0096 (15)	0.0022 (16)	0.0021 (13)
C2	0.037 (2)	0.0160 (17)	0.0103 (15)	−0.0053 (15)	−0.0003 (16)	0.0024 (13)
N2	0.0319 (18)	0.0090 (13)	0.0093 (13)	−0.0019 (11)	0.0033 (13)	0.0010 (10)
C3	0.039 (2)	0.0161 (17)	0.0119 (16)	−0.0099 (15)	0.0021 (16)	0.0006 (13)
C4	0.036 (2)	0.0161 (17)	0.0110 (15)	−0.0066 (15)	−0.0009 (16)	0.0028 (13)
N11	0.0312 (18)	0.0083 (13)	0.0119 (13)	0.0028 (11)	0.0100 (13)	0.0027 (10)
C11	0.036 (2)	0.0137 (17)	0.0241 (18)	0.0075 (15)	0.0172 (17)	0.0096 (14)
C12	0.036 (2)	0.0147 (16)	0.0220 (17)	0.0080 (15)	0.0182 (17)	0.0087 (14)
N12	0.0331 (18)	0.0079 (13)	0.0129 (14)	0.0033 (11)	0.0126 (14)	0.0015 (10)
C13	0.041 (2)	0.0177 (18)	0.0257 (19)	0.0137 (16)	0.0198 (19)	0.0117 (15)
C14	0.039 (2)	0.0179 (18)	0.0278 (19)	0.0093 (16)	0.0216 (18)	0.0111 (15)
N21	0.0311 (18)	0.0192 (16)	0.0204 (15)	−0.0023 (14)	0.0056 (16)	−0.0001 (12)
C21	0.034 (4)	0.032 (4)	0.040 (4)	0.000 (3)	0.014 (3)	−0.027 (3)
O21	0.041 (4)	0.137 (8)	0.152 (8)	−0.033 (4)	0.031 (5)	−0.114 (7)
O21'	0.027 (7)	0.061 (11)	0.055 (9)	0.012 (7)	−0.016 (8)	−0.038 (8)
C21'	0.040 (12)	0.004 (8)	0.023 (9)	0.001 (7)	0.003 (9)	0.004 (6)
N31	0.0318 (18)	0.0171 (16)	0.0183 (15)	0.0012 (13)	0.0099 (15)	−0.0002 (12)
C31	0.038 (4)	0.018 (3)	0.016 (3)	0.007 (3)	0.001 (3)	−0.010 (3)
O31	0.052 (4)	0.089 (5)	0.055 (4)	0.036 (4)	−0.031 (4)	−0.053 (4)

### Geometric parameters (Å, °)

**Table:**

Co1—N21	2.039 (3)	N11—C11	1.337 (5)
Co1—N31	2.059 (3)	C11—C12	1.390 (5)
Co1—N11	2.191 (3)	C11—H11	0.9500
Co1—N2^i^	2.193 (3)	C12—N12	1.333 (5)
Co1—N12^ii^	2.197 (3)	C12—H12	0.9500
Co1—N1	2.200 (3)	N12—C13	1.332 (5)
N1—C1	1.324 (5)	N12—Co1^iv^	2.197 (3)
N1—C4	1.339 (5)	C13—C14	1.397 (5)
C1—C2	1.394 (5)	C13—H13	0.9500
C1—H1	0.9500	C14—H14	0.9500
C2—N2	1.337 (5)	N21—C21	1.151 (8)
C2—H2	0.9500	N21—C21'	1.18 (2)
N2—C3	1.338 (5)	C21—O21	1.206 (9)
N2—Co1^iii^	2.193 (3)	O21'—C21'	1.19 (3)
C3—C4	1.395 (5)	N31—C31'	1.04 (2)
C3—H3	0.9500	N31—C31	1.182 (8)
C4—H4	0.9500	C31—O31	1.195 (9)
N11—C14	1.334 (5)	C31'—O31'	1.24 (4)
			
N21—Co1—N31	179.45 (13)	N1—C4—H4	119.2
N21—Co1—N11	90.19 (12)	C3—C4—H4	119.2
N31—Co1—N11	89.76 (12)	C14—N11—C11	116.8 (3)
N21—Co1—N2^i^	90.23 (12)	C14—N11—Co1	121.8 (2)
N31—Co1—N2^i^	89.23 (12)	C11—N11—Co1	121.2 (2)
N11—Co1—N2^i^	90.53 (11)	N11—C11—C12	121.5 (3)
N21—Co1—N12^ii^	90.19 (12)	N11—C11—H11	119.2
N31—Co1—N12^ii^	90.35 (12)	C12—C11—H11	119.2
N11—Co1—N12^ii^	89.45 (10)	N12—C12—C11	121.6 (3)
N2^i^—Co1—N12^ii^	179.58 (12)	N12—C12—H12	119.2
N21—Co1—N1	90.57 (12)	C11—C12—H12	119.2
N31—Co1—N1	89.49 (12)	C13—N12—C12	117.1 (3)
N11—Co1—N1	178.55 (11)	C13—N12—Co1^iv^	121.0 (2)
N2^i^—Co1—N1	90.70 (10)	C12—N12—Co1^iv^	121.9 (2)
N12^ii^—Co1—N1	89.32 (10)	N12—C13—C14	121.3 (3)
C1—N1—C4	116.7 (3)	N12—C13—H13	119.3
C1—N1—Co1	120.9 (2)	C14—C13—H13	119.3
C4—N1—Co1	122.5 (2)	N11—C14—C13	121.6 (3)
N1—C1—C2	122.1 (3)	N11—C14—H14	119.2
N1—C1—H1	118.9	C13—C14—H14	119.2
C2—C1—H1	118.9	C21—N21—C21'	34.7 (8)
N2—C2—C1	121.5 (3)	C21—N21—Co1	153.4 (5)
N2—C2—H2	119.3	C21'—N21—Co1	150.6 (11)
C1—C2—H2	119.3	N21—C21—O21	177.2 (8)
C2—N2—C3	116.5 (3)	N21—C21'—O21'	174 (2)
C2—N2—Co1^iii^	120.1 (2)	C31'—N31—C31	24.8 (10)
C3—N2—Co1^iii^	123.4 (2)	C31'—N31—Co1	163.3 (12)
N2—C3—C4	121.6 (3)	C31—N31—Co1	150.1 (5)
N2—C3—H3	119.2	N31—C31—O31	178.7 (10)
C4—C3—H3	119.2	N31—C31'—O31'	174 (2)
N1—C4—C3	121.6 (3)		

Symmetry codes: (i) *x*, −*y*+1, *z*−1/2; (ii) *x*, −*y*+2, *z*+1/2; (iii) *x*, −*y*+1, *z*+1/2; (iv) *x*, −*y*+2, *z*−1/2.
